# Impact of Positive Lymph Nodes and Resection Margin Status on the Overall Survival of Patients with Resected Perihilar Cholangiocarcinoma: The ENSCCA Registry

**DOI:** 10.3390/cancers14102389

**Published:** 2022-05-12

**Authors:** Lynn E. Nooijen, Jesus M. Banales, Marieke T. de Boer, Chiara Braconi, Trine Folseraas, Alejandro Forner, Waclaw Holowko, Frederik J. H. Hoogwater, Heinz-Josef Klümpen, Bas Groot Koerkamp, Angela Lamarca, Adelaida La Casta, Flora López-López, Laura Izquierdo-Sánchez, Alexander Scheiter, Kirsten Utpatel, Rutger-Jan Swijnenburg, Geert Kazemier, Joris I. Erdmann

**Affiliations:** 1 Department of Surgery, Amsterdam UMC—Location Vrije Universiteit Amsterdam, 1081 HV Amsterdam, The Netherlands; l.e.nooijen@amsterdamumc.nl (L.E.N.); g.kazemier@amsterdamumc.nl (G.K.); 2 Cancer Center Amsterdam, Cancer Treatment and Quality of Life, 1081 HV Amsterdam, The Netherlands; h.klumpen@amsterdamumc.nl (H.-J.K.); r.j.swijnenburg@amsterdamumc.nl (R.-J.S.); 3 Department of Liver and Gastrointestinal Diseases, Biodonostia Health Research Institute, Donostia University Hospital, University of the Basque Country (UPV/EHU), 20014 San Sebastián, Spain; jesus_banales@yahoo.es (J.M.B.); laura.izquierdo@biodonostia.org (L.I.-S.); 4 National Institute for the Study of Liver and Gastrointestinal Diseases, CIBERehd, “Instituto de Salud Carlos III” (ISCIII), 28029 Madrid, Spain; 5 Department of Biochemistry and Genetics, School of Sciences, University of Navarra, 31080 Pamplona, Spain; 6 Ikerbasque, Basque Foundation for Science, 48009 Bilbao, Spain; 7 Section of HPB Surgery & Liver Transplantation, Department of Surgery, University Medical Center Groningen, University of Groningen, 9713 GZ Groningen, The Netherlands; m.t.de.boer@umcg.nl (M.T.d.B.); f.j.h.hoogwater@umcg.nl (F.J.H.H.); 8 The Royal Marsden NHS Trust, London SW3 6JJ, UK; chiarabraconi@gmail.com; 9 Norwegian PSC Research Center and Section of Gastroenterology, Department of Transplantation Medicine, Division of Surgery, Inflammatory Medicine and Transplantation, Oslo University Hospital Rikshospitalet, 0372 Oslo, Norway; trine.folseraas@medisin.uio.no; 10 BCLC Group, Liver Unit, Hospital Clinic Barcelona, IDIBAPS, CIBEREHD, University Barcelona, 08036 Barcelona, Spain; aforner@clinic.cat; 11 Department of General, Transplant and Liver Surgery, Medical University of Warsaw, 02-091 Warsaw, Poland; holowko.waclaw@gmail.com; 12 Department of Medical Oncology, Amsterdam UMC—Location University of Amsterdam, 1105 AZ Amsterdam, The Netherlands; 13 Department of Surgery, Erasmus MC Cancer Institute, 3015 GD Rotterdam, The Netherlands; b.grootkoerkamp@erasmusmc.nl; 14 Department of Medical Oncology, The Christie NHS Foundation Trust, Wilmslow Road, Manchester M20 4BX, UK; angela.lamarca@christie.nhs.uk; 15 Department of Medical Oncology, Biodonostia Health Research Institute, Donostia University Hospital, University of the Basque Country (UPV/EHU), 20014 San Sebastián, Spain; delaida.lacastamunoa@osakidetza.eus; 16 Department of Medical Oncology, Hospital Universitario 12 de Octubre, 28041 Madrid, Spain; flora_989@hotmail.com; 17 Institute of Pathology, University of Regensburg, 93053 Regensburg, Germany; alexander.scheiter@klinik.uni-regensburg.de (A.S.); kirsten.utpatel@klinik.uni-regensburg.de (K.U.); 18 Department of Surgery, Amsterdam UMC—Location University of Amsterdam, 1105 AZ Amsterdam, The Netherlands

**Keywords:** perihilar cholangiocarcinoma, lymph nodes, resection margin, overall survival, recurrence-free survival

## Abstract

**Simple Summary:**

Lymph node metastasis and positive resection margins have been reported to be major determinants of overall survival (OS) and poor recurrence-free survival (RFS) for patients after resection for perihilar cholangiocarcinoma (pCCA). The aim of current study was to assess the prognostic value of positive lymph nodes and resection margin status on OS. We found a major negative effect of positive lymph nodes on survival and recurrence, which, surprisingly, was independent of margin status. ECOG performance status, positive lymph nodes, and the grade of tumor differentiation were found as the main independent indicators of oncological outcome. This suggests that tumor biology and performance status have a higher impact on survival than extended or radical surgery.

**Abstract:**

Background: Lymph node metastasis and positive resection margins have been reported to be major determinants of overall survival (OS) and poor recurrence-free survival (RFS) for patients who underwent resection for perihilar cholangiocarcinoma (pCCA). However, the prognostic value of positive lymph nodes independently from resection margin status on OS has not been evaluated. Methods: From the European Cholangiocarcinoma (ENSCCA) registry, patients who underwent resection for pCCA between 1994 and 2021 were included in this retrospective cohort study. The primary outcome was OS stratified for resection margin and lymph node status. The secondary outcome was recurrence-free survival. Results: A total of 325 patients from 11 different centers and six European countries were included. Of these, 194 (59.7%) patients had negative resection margins. In 113 (34.8%) patients, positive lymph nodes were found. Lymph node status, histological grade, and ECOG performance status were independent prognostic factors for survival. The median OS for N0R0, N0R1, N+R0, and N+R1 was 38, 30, 18, and 12 months, respectively (*p* < 0.001). Conclusion: These data indicate that in the presence of positive regional lymph nodes, resection margin status does not determine OS or RFS in patients with pCCA. Achieving negative margins in patients with positive nodes should not come at the expense of more extensive surgery and associated higher mortality.

## 1. Introduction

Radical surgical resection is still the only treatment with a chance of long-term survival for patients with perihilar cholangiocarcinoma (pCCA). Radical (R0) resection rates range from 60 to 90%, which is associated with a 5-year overall survival (OS) up to 60%, while a margin positive (R1) resection is associated with a 5-year OS of less than 20% [1,2,3,4,5]. On the other hand, the prognosis of patients with resected pCCA is also significantly determined by the presence of lymph node metastases [1,6,7].

At exploration, about 35% of patients have loco-regional positive lymph nodes, which is associated with a 5-year OS of less than 20% versus 55% in case of negative lymph nodes [1,6,7]. During resection, the retrieval of a low number of less than four lymph nodes has been reported to negatively influence survival, possibly due to the understaging of unresected positive nodes [8]. In addition, a recent systematic review found that an extended lymphadenectomy does not lead to a survival benefit [9].

Nevertheless, a high number of involved lymph nodes has been reported to be a major determinant of OS and an indicator of poor recurrence-free survival (RFS) [8,10,11]. Buettner et al. concluded that the actual survival benefit of resection in patients with positive lymph nodes is shorter than seven months and should be weighed against considerable postoperative morbidity and mortality [12].

The updated AJCC guidelines (8th edition, 2017) distinguish between the number (*n* ≤ 3 or *n* > 3) instead of the location of regional positive lymph nodes [13,14]. Several studies investigated the value of this new staging system and found a slightly improved prognostic predictability, implying that the amount of involved lymph nodes is more predictive for survival outcome than their location [15,16,17].

In clinical practice, surgeons are often confronted with one or more positive lymph nodes at frozen section analysis in a patient undergoing surgical exploration with the intention to undergo an oftentimes major hepatobiliary resection. The technical feasibility of a potential margin-free resection and concomitant morbidity and mortality should be evaluated in the light of potential oncological outcomes. Until now, the relationship between positive lymph nodes and resection margins and their independent influence on OS remained unclear, making the question of whether patients with positive lymph nodes benefit from extended resections to obtain negative margins hard to answer. Therefore, we performed a retrospective cohort study, in cooperation with the ENSCCA Registry, to determine the impact of positive lymph nodes and resection margin status on the OS of patients after resected pCCA. 

## 2. Materials and Methods

### 2.1. ENSCCA Registry

The ENSCCA registry, endorsed by the European Network for the Study of Cholangiocarcinoma (ENSCCA), is an international, multicenter, and observational retrospective and prospective study including patients with histologically or radiologically proven cholangiocarcinoma (intrahepatic, perihilar, and distal cholangiocarcinoma). This registry includes several different variables regarding surgical and oncological outcomes. The ENSCCA registry protocol was approved by the Ethic Committee of Euskadi, Spain (Code: PI2016137) and the Ethic Committee of the Amsterdam UMC (location AMC), the Netherlands (W20_050), as coordinating Centers. Additionally, each participating center obtained local ethical approval (or equivalent).

### 2.2. Included Patients

From the ENSCCA registry, patients with pCCA who underwent surgery including diagnostic laparoscopy, explorative laparotomy, liver transplantation, or liver resection were identified. Only patients from centers that had registered a minimum of 10 patients undergoing resection for pCCA were included. This was in order to secure centers with expertise in treating patients with pCCA for inclusion. Eligible centers were asked to complete potentially missing key variables (e.g., resection margin, lymph node status, and last date of follow-up) or to check discrepant variables. For the analysis, only patients who received curative-intent resection were included. 

### 2.3. Outcomes 

The primary outcome was to assess the impact of the resection margin and lymph node status on OS. The secondary outcome was the impact of resection margin and lymph node status on recurrence-free survival (RFS).

### 2.4. Statistical Analysis

Descriptive statistics were used to summarize the data. For continuous variables with normal distribution, the data were presented as mean ± standard deviation (SD) and for non-normal distributions, as median and interquartile range (IQR). Comparisons between the groups (N0 and N+) were analyzed using chi-square tests for proportions, Mann–Whitney U for medians, and unpaired t-tests for means. The OS was defined as the time (in months) after surgery to death or last follow-up, and the median OS was estimated using the Kaplan–Meier method. The RFS was defined as the time (in months) after surgery to recurrence or last medical visit. Survival curves were compared using the log-rank test. The reversed Kaplan–Meier-based method was used to calculate median follow-up. Univariable and multivariable logistic regression were used to examine the relationship between the presence of positive lymph nodes as a dependent variable and the possible predictors as independent variables. Therefore, the following factors were included: age, ECOG, and tumor size > 2.5 cm. Factors with *p*-values < 0.2 at univariable analysis were included in the multivariable analysis. The independent predictors of survival determined prior to conducting the study (i.e., age, ECOG, tumor size > 2.5 cm, carbohydrate antigen 19.9 (CA19.9), lymph node status (AJCC 8th), margin status, and tumor differentiation [8,11]) were analyzed using uni- and multivariable Cox regression analysis. In addition, the independent predictors of recurrence, also determined prior to conducting the study (tumor size > 2.5 cm, T status (AJCC 8th), lymph node status (AJCC 8th), resection margin, and tumor differentiation) were analyzed using uni- and multivariable Cox regression analysis [10,18]. Factors with *p*-values < 0.1 at univariable analysis were included in the multivariable analysis. A two-sided *p*-value of < 0.05 was considered to indicate statistical significance. The data were analyzed using IBM SPSS statistics, version 25.0 (IBM Corp, Armonk, NY, USA). Survival curves were displayed using GraphPad Prism 8. 

## 3. Results

### 3.1. Baseline Characteristics

**Figure 1:**
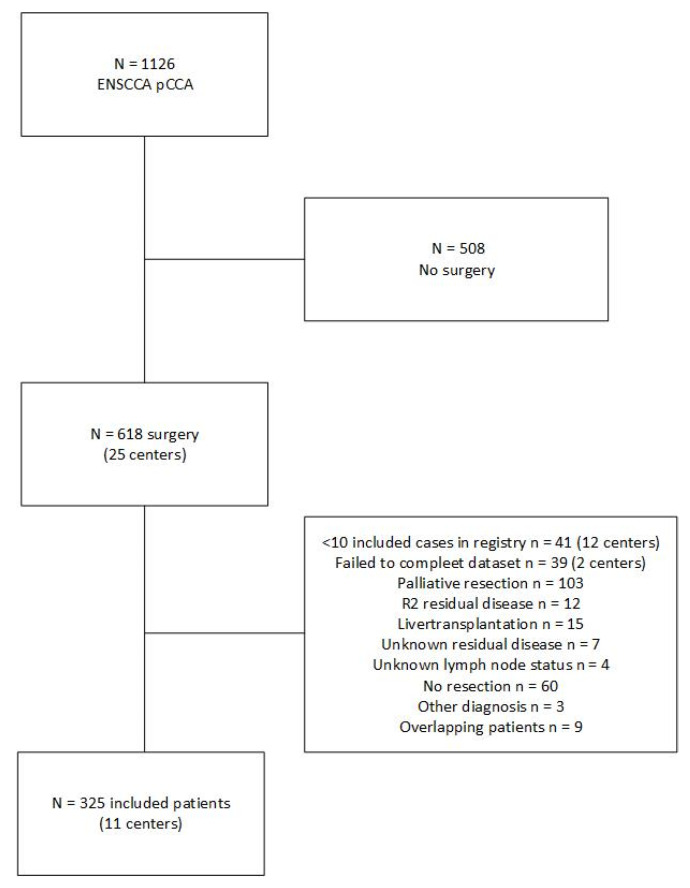
Flow diagram of included patients.

A total of 1126 patients with pCCA were extracted from the registry; from these, 618 (55%) patients underwent any type of surgery. Forty-one patients were excluded as these were from centers that registered fewer than ten patients. Thirty-nine patients were excluded because of missing information. From this dataset, patients undergoing palliative resection (*n* = 103), undergoing liver transplantation (*n* = 15), who had R2 residual disease (*n* = 12), unknown residual disease (*n* = 7), unknown lymph node status (*n* = 4), who had no resection (*n* = 60), other diagnosis (*n* = 3), or were double in the registry (*n* = 9) were excluded, leaving a total of 325 patients from 11 different centers in six countries. A flowchart is presented in Figure 1. 

A total of 206 out of the 325 patients (63.4%) were male and the median age at surgery was 64 (58–71) years. The median Bismuth–Corlette classification was IIIb (20.6%). The median preoperative tumor size was 2.5 (1.9–3.7) cm. Neoadjuvant therapy, consisting of radiotherapy 5 × 5 Gy, was administered to 26 (8.0%) patients.

Out of the 325 patients, 194 (59.7%) had undergone an R0 resection. In 113 (34.8%) patients, positive lymph nodes were found. In the latter group, the median number of retrieved lymph nodes was 5 (IQR: 3–7) and the median number of positive lymph nodes was 2 (IQR: 1–3), as shown in Table 1. When selected for resection margin and lymph node status, the following groups were formed: 138 patients with N0R0, 74 patients with N0R1, 56 patients with N+R0, and 57 patients with N+R1. Following the AJCC 8th edition, N2 disease was found in 20 (17.7%) patients and N1 in 93 (82.3%) patients. The location of the lymph nodes was only mentioned in 14 patients (12.4%); therefore, we were not able to include the location or specific lymph node stations in the analysis. 

### 3.2. N+ versus N0

When comparing the included patients for lymph node status, the age at surgery, size and extent of the tumor, number of resected lymph nodes, resection margin, and recurrence were significantly different (Table 1). Patients with positive lymph nodes were approximately four years younger and presented with a higher tumor (T) stage. In these patients, a higher median number of lymph nodes were dissected in contrast to lymph node-negative patients (N+: 5 vs. N0: 4). The R1 resection margin (N+: 50.4% vs. N0: 34.9%) and higher tumor recurrence rate were associated with N+ status (N+: 54.9% vs. N0: 42.0%). On univariable logistic regression analyses, age (OR: 0.98 (95% CI 0.96–1.00)) and tumor size > 2.5 cm (OR: 1.62 (95% CI 0.92–2.85)) were independently associated with positive lymph nodes. However, on multivariable logistic regression analysis, these factors were not independently associated with positive lymph nodes (Table S1). 

### 3.3. Overall Survival

Out of the 325 patients, 114 patients were alive and 211 had died at final follow-up. The median follow-up of patients alive at last medical visit was 59 months (95% CI 57.6–60.4). Out of the 325 patients, 59 (18%) patients received adjuvant (chemo)therapy. This concerned chemotherapy in 52 (16%) patients, chemo–radiotherapy in four (1%), and radiotherapy in one (0.3%) patient. For two patients, this was unknown. 

**Figure 2:**
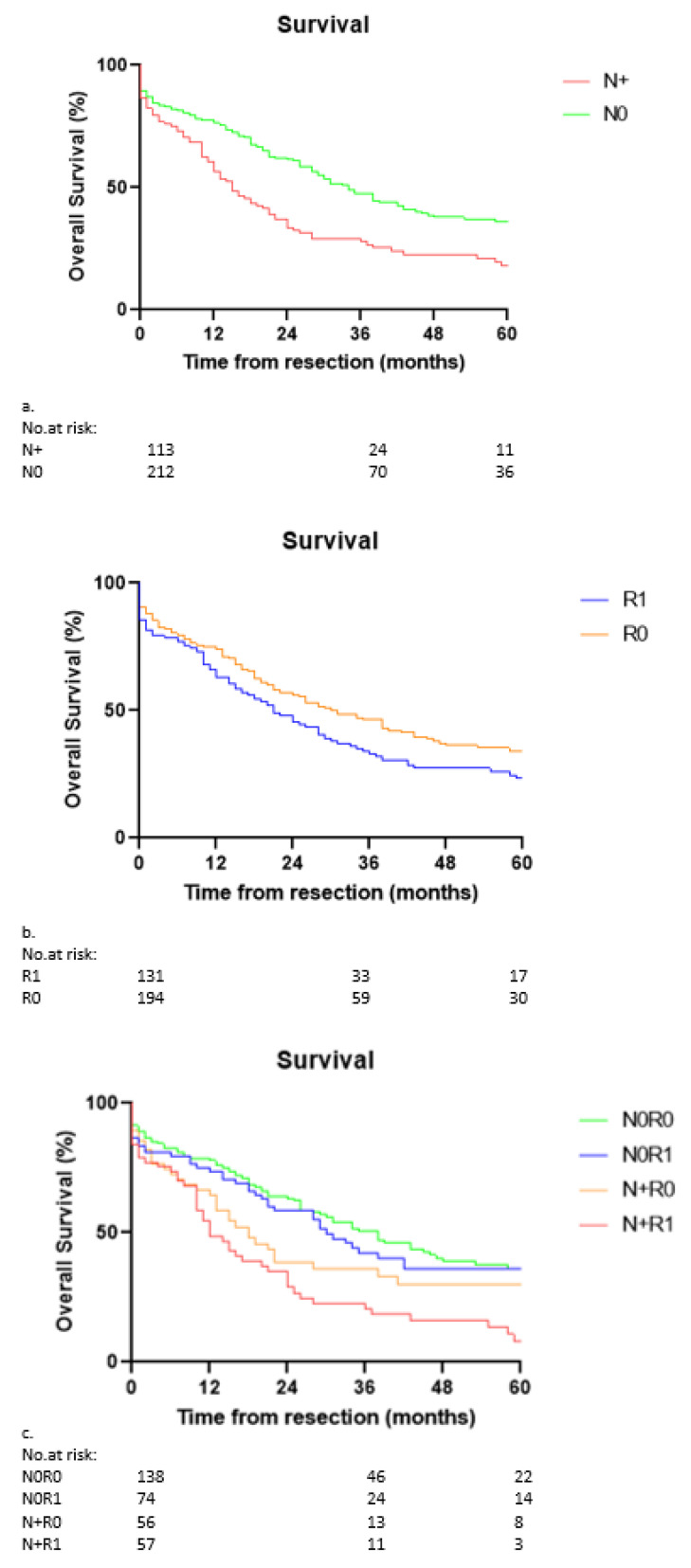
(**a**): Kaplan–Meier curve for overall survival and lymph node-negative (N0) and lymph node-positive (N+) patients. Only the first 5 years are displayed. Median OS was 34 months (95% CI 27.9–40.1) for the N0 versus 15 months (95% CI 10.6–19.4) for the N1 patients (*p* < 0.001). (**b**): Kaplan–Meier curve for overall survival of resection margin-negative (R0) and resection margin-positive (R1) patients. Only the first 5 years are displayed. Median OS was 31 months (95% CI 23.0–39.0) for R0 versus 21 months (95% CI 14.9–27.2) for R1 patients (*p* = 0.037). (**c**): Kaplan–Meier curve for overall survival of N0R0, N0R1, N+R0, and N+R1 patients. Only the first 5 years are displayed. Median OS was 38 months (95% CI 28.0–48.0) for N0R0, 30 months (95% CI 19.8–40.2) for N0R1, 18 months (95% CI 10.7–25.3) for N+R0, and 12 months (95% CI 7.7–16.3) for the N+R1 patients (*p* < 0.001). N0R0 vs. N+R0: *p* = 0.064, N0R1 vs. N+R1: *p* < 0.001, N+R0 vs. N+R1: *p* = 0.061, N0R0 vs. N0R1: *p* = 0.61.

The median and 5-year OS for the whole group was 26 months (95% CI 21.0–31.0) and 29%, respectively. The median and 5-year OS divided for lymph node status were 34 months (95% CI 27.9–40.1) and 36% for N0 patients, and 15 months (95% CI 10.6–19.4) and 18% for N+ patients (*p* < 0.001; Figure 2a). The median and 5-year OS divided for resection margin was 31 months (95% CI 23.0–39.0) and 34% for R0 disease, and 21 months (95% CI 14.9–27.2) and 23% for R1 disease (*p* < 0.05; Figure 2b). Including both resection margin and lymph node status (N0R0, N0R1, N+R0 and N+R1), the median and 5-year OS were 38 months (95% CI 28.0–48.0) and 36%, 30 months (95% CI 19.8–40.2) and 36%, 18 months (95% CI 10.7–25.3) and 30%, and 12 months (95% CI 7.7–16.3) and 8%, respectively (*p* < 0.001, Figure 2c). The comparison per status was as follows: N0R0 vs. N+R0: *p* = 0.064, N0R1 vs. N+R1: *p* < 0.001, N+R0 vs. N+R1: *p* = 0.061, N0R0 vs. N0R1: *p* = 0.61.

The prognostic factors for OS were analyzed using a uni- and multivariable Cox regression analysis. On univariable analysis, ECOG performance status, lymph node status, resection margin, and tumor differentiation grade were negatively associated with OS (*p* < 0.1). After multivariable Cox regression analysis, ECOG performance status (ECOG 2: HR 2.95 (95% CI 1.55–5.63)), positive lymph nodes (N1: HR 1.92 (95% CI 1.29–2.85)), and tumor differentiation (poorly differentiated: HR 1.80 (95% CI 1.14–2.84)) were identified as independent prognostic factors for OS (*p* < 0.05; Table 2). 

### 3.4. Recurrence 

During follow-up, 156 (48%) patients developed recurrence. The recurrence status at final follow-up was unknown for 52 (16%) patients. Recurrence occurred most often distant from the primary tumor site (*n* = 63, 41.7%), being mostly found in the peritoneum (*n* = 28, 49.1%; Table 1). From the patients who had recurrence, 32 (21%) patients received and 120 (77%) patients did not receive adjuvant (chemo)therapy. For four patients, this was unknown. 

**Figure 3:**
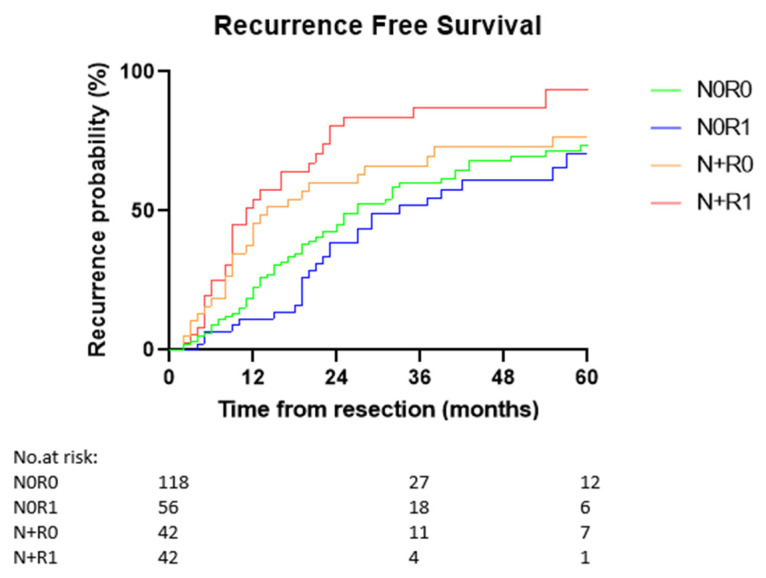
Estimated cumulative recurrence probability of N0R0, N0R1, N+R0, and N+R1 patients. Only the first 5 years are displayed. The median recurrence-free survival was 27 months (95% CI 20.4–33.6) for N0R0, 33 months (95% CI 19.5–46.5) for N0R1, 14 months (95% CI 7.4–20.6) for N+R0, and 11 months (95% CI 5.7–16.3) for the N+R1 patients (*p* < 0.001). N0R0 vs. N+R0: *p* = 0.151, N0R1 vs. N+R1: *p* < 0.001, N+R0 vs. N+R1: *p* = 0.109, N0R0 vs. N0R1: *p* = 0.331.

The overall median recurrence-free survival was 23 months (95% CI 19.2–26.8). The recurrence-free survival divided for resection margin and lymph node status (N0R0, N0R1, N+R0, and N+R1) was 27 months (95% CI 20.4–33.6), 33 months (95% CI 19.5–46.5), 14 months (95% CI 7.4–20.6), and 11 months (95% CI 5.7–16.3), respectively (*p* < 0.001; Figure 3). The comparison per status was: N0R0 vs. N+R0: *p* = 0.151, N0R1 vs. N+R1: *p* < 0.001, N+R0 vs. N+R1: *p* = 0.109, N0R0 vs. N0R1: *p* = 0.331.

The prognostic factors for recurrence-free survival were also analyzed using a uni- and multivariable Cox regression analysis. On univariable analysis, lymph node status, T stage (AJCC 8th), and tumor differentiation were negatively associated with OS (*p* < 0.1). After multivariable Cox regression analysis, positive lymph nodes (N1: HR 2.62 (95% CI 1.68–4.08)) and tumor differentiation (poorly differentiated: HR 1.65 (95% CI 1.05–2.58)) were identified as independent prognostic factors for recurrence (*p* < 0.05; Table 3).

## 4. Discussion

This study shows that for patients who undergo a resection for pCCA, positive lymph nodes have a major negative effect on recurrence and survival. Surprisingly, this was independent of resection margin status. In addition, ECOG performance status, positive lymph nodes, and the grade of tumor differentiation were found as the main independent indicators of oncological outcome. These findings suggest that tumor biology and performance status have a higher impact on survival than extended or radical surgery. 

Several studies describe the outcomes of patients with resected pCCA. However, most of these studies are single-center or single-country studies and often only include small numbers of patients. The present analysis is the first study from the ENSCCA registry addressing resected pCCA. The registry represents a large cohort, with patients from multiple expert centers across Europe. 

Previous studies reported comparable differences between the survival rates for patients with and without lymph node involvement [4,6,19]. For patients with locally advanced disease who do not undergo resection, median OS rates of 12 months are reported. With the OS for N+ between 12 and 18 months and the mortality rate of 23% found in this study in mind, the question of whether node-positive patients actually benefit from this form of extensive surgery remains [12].

The multivariable analyses performed in this study showed that the resection margin itself was not an independent prognostic factor for OS. It is known that defining negative resection margins is difficult in patients with pCCA. In order to properly assess R0 disease, all resection planes must be investigated correctly [20]. However, there is currently no standardized protocol, causing differences between different hospitals. As a consequence, it is likely that the R1 rate is underestimated in our study.

This study shows several other remarkable findings. A four-year age difference between lymph node-positive and lymph node-negative patients was found. Besides this, the T stage was higher in the lymph node-positive group. These differences could have two explanations: the first would be that young patients are prone to having a more aggressive tumor with advanced tumor characteristics, including higher tumor stages and positive lymph nodes. A second and more likely explanation would be that a surgeon is more inclined to perform a resection in young patients, whereas in older and more fragile patients, the resection is not performed due to a less favorable risk profile. 

The median number of lymph nodes that were retrieved in lymph node-positive patients was higher than in lymph node-negative patients. This may have resulted in an understaging of patients with negative lymph nodes. This is supported by the conclusion of a recent systematic review stating that a cut-off of five lymph nodes should be used to avoid understaging [9]. On the other hand, in older or more fragile patients, a surgeon may sometimes sample more lymph nodes to more accurately stage and to avoid futile major surgery. However, this is not reflected in our data due to the fact that these patients may not have undergone a resection, but does support the difference in age found in our study. 

The high impact of positive lymph nodes on survival and recurrence found in this study raises the question of whether standardized lymphadenectomy with frozen section analysis should be performed in order to “protect” fragile patients from a futile resection with high 90-day mortality rates, early recurrence, and poor survival. The same systematic review mentioned earlier also found that an extended lymphadenectomy is not associated with survival benefit. Therefore, the main goal of lymph node dissection is to accurately stage patients with pCCA, especially in more advanced-stage tumors, and to balance the risk and benefit of major surgery [21,22]. However, a surgeon should be aware of the possibility of false-negative frozen section results, especially in large lymph nodes with large amounts of fat in or around the node. 

The results of this study are in line with the results of liver transplant for pCCA [23]. For liver transplant, positive nodes are currently considered a strict contraindication due to the poor oncological outcomes in these patients. Interestingly, these patients undergo a total hepatectomy, the most radical form of resection of the bile ducts. This further underlines the main point of the present study: the biology (reflected by positive nodes) of the tumor probably indicates poor outcome irrespective of the extent of the resection [24].

This study needs to be seen in the light of some limitations. First, the registry does not include the type of resection (e.g., hemihepatectomy). The left (extended) hemihepatectomy was probably the most-performed resection, as most patients had a Bismuth–Corlette type IIIb, although this is still speculation. Post-operative complications were not included in the registry; therefore, we could only include mortality and not morbidity in the analysis. In addition, angio-invasion and perineural growth were not reported in this registry. From the available literature, it is known that both factors are important prognostic determinants [11]. Adjuvant chemotherapy was not included in this uni- and multivariable analysis due to the fact that very few patients received adjuvant chemotherapy. Moreover, the influence on survival is still uncertain. To avoid any possible selection bias, this factor was not included in the analysis.

Besides the shortcomings of the registry, it is important to mention that centers use their own experience and guidelines when it comes to the treatment of pCCA. Therefore, the selection, treatment, and outcomes may differ from center to center. In addition, this study included patients from 1994–2021, which could have introduced bias, although the focus of the included patients was on the latter years. In addition, although this study includes a relatively high number of patients, the study may be underpowered in terms of identifying small effects. This is clearly illustrated when we compare our data to the recently published study by Izquierdo et al. [25]. They found CA19.9 as an independent prognostic factor for OS, which we were not able to confirm. In addition, they found a survival difference between N0R0 and N0R1 resections, possibly caused by the fact that their study included all subtypes of cholangiocarcinoma.

The results of the present study indicate that other (pre)operative strategies to assess lymph nodes are needed. One could be the addition of 18F-fluorodeoxyglucose positron emission tomography (18FDG-PET) to the pre-operative work-up. A systematic review found a pooled sensitivity and specificity of 88.4% and 69.1%, respectively, for the detection of lymph node metastasis [26]. In addition to the 18FDG-PET strategy, staging (robotic) laparoscopy could confirm these findings [27,28]. Although staging laparoscopy is only advised in selected patients, there might be an important role for robotic surgery, as this seems to increase the options to minimally invasively retrieve nodes at the celiac trunk and retro-pancreatic area. In addition, during staging laparoscopy, although not specifically investigated in patients with pCCA, indocyanine green or more tumor-specific tracers might have an added value in optimizing lymph node dissection [29]. Thus, due to the fact that most of these strategies have not been investigated in patients with pCCA, this is a clear area for future research. 

## 5. Conclusions

The results of the present study suggest that, in the presence of positive lymph nodes, the resection margin does not determine overall or recurrence-free survival in patients with pCCA. Therefore, achieving negative margins in patients with positive nodes should not come at the expense of more extensive surgery and associated mortality. 

## Figures and Tables

**Table 1:** Baseline and postoperative characteristics according to resection margin and lymph node status.

	Total	N0	N+	*p*-Value
Patients	*n* = 325	*n* = 212	*n* = 113	
Age at surgery (median)	64 (58–71)	67 (58.3–72.0)	63 (57–69)	0.028
Male (%)	206 (63.4%)	135 (63.7%)	71 (62.8%)	0.880
ECOG performance status				0.901
0	143 (44.0%)	96 (45.3%)	47 (41.6%)	
1	81 (24.9%)	51 (24.1%)	30 (26.5%)	
2	17 (5.2%)	12 (5.7%)	5 (4.4%)	
3	4 (1.2%)	3 (1.4%)	1 (0.9%)	
unknown	80 (24.6%)	50 (23.6%)	30 (26.5%)	
Bismuth–Corlette				0.931
I	18 (5.5%)	11 (5.2%)	7 (6.2%)	
II	20 (6.2%)	14 (6.6%)	6 (5.3%)	
IIIa	40 (12.3%)	26 (12.3%)	14 (12.4%)	
IIIb	67 (20.6%)	47 (22.2%)	20 (17.7%)	
IV	49 (15.1%)	31 (14.6%)	18 (15.9%)	
Unknown	131 (40.3%)	83 (39.2%)	48 (42.5%)	
CA19.9	67 (11–313)	57 (10–252)	174 (13–604)	0.070
Bilirubin	12 (2–116)	12 (2–131)	13 (3–106)	0.706
Preoperative tumor size (cm) (median) (N = 221)	2.5 (1.9–3.7)	2.3 (1.7–3.6)	2.8 (2.1–4.0)	0.022
Neoadjuvant therapy ±	26 (8.0%)	17 (14.8%)	9 (16.4%)	0.789
Size and extent (T)				0.011
T1	20 (6.2%)	18 (8.5%)	2 (1.8%)	
T2a	80 (24.8%)	59 (28.0%)	21 (18.8%)	
T2b	93 (28.8%)	60 (28.4%)	33 (29.5%)	
T3	92 (28.5%)	50 (23.7%)	42 (37.5%)	
T4	31 (9.6%)	18 (8.5%)	13 (11.6%)	
Tx	7 (2.2%)	6 (2.8%)	1 (0.9%)	
Regional lymph nodes (N #)				-
N0	212 (65.2%)	212 (100%)	0	
N1	93 (28.6%)	0	93 (82.3%)	
N2	20 (6.2%)	0	20 (17.7%)	
Total number of resected lymph nodes (median)	4 (2–7)	4 (2–6)	5 (3–7)	0.024
Number of positive lymph nodes (median) (*n* = 103)	2 (1–3)	-	2 (1–3)	-
Resection margin				0.007
R0	194 (59.7%)	138 (65.1%)	56 (49.6%)	
R1	131 (40.3%)	74 (34.9%)	57 (50.4%)	
Differentiation grade				0.550
Grade 1: well differentiated	40 (12.3%)	29 (13.7%)	11 (9.7%)	
Grade 2: moderately differentiated	150 (46.2%)	93 (43.9%)	57 (50.4%)	
Grade 3: poorly differentiated	62 (19.1%)	43 (20.3%)	19 (16.8%)	
Not available	73 (22.5%)	47 (22.2%)	26 (23.0%)	
Adjuvant therapy	59 (18.2%)	34 (17.3%)	23 (23.1%)	0.213
Recurrence *	151 (46.5%)	89 (42.0%)	62 (54.9%)	0.003
Location of recurrence				0.990
Local recurrence	54 (35.8%)	31 (34.8%)	23 (37.1%)	
Liver	31 (20.5%)	18 (20.2%)	13 (21.0%)	
Distant	63 (41.7%)	38 (42.7%)	25 (40.3%)	
Unknown	3 (2.0%)	2 (2.2%)	1 (1.6%)	
90-day mortality	70 (21.5%)	44 (20.8%)	26 (23.0%)	0.638

*p*-values based on complete case analysis unless unknown is displayed. * Censored at 5-year follow-up. # According to AJCC 8th edition. ± Radiotherapy 5 × 5 Gy.

**Table 2:** Uni- and multivariable Cox regression analysis for overall survival.

	Univariable Analysis		Multivariable Analysis	
	HR (95% CI)	*p*-Value ^$^	HR (95% CI)	*p*-Value ^#^
Age at surgery	1.01 (0.99–1.02)	0.287		
ECOG performance status				
ECOG 0	Reference		Reference	
ECOG 1	1.21 (0.84–1.74)	0.297	1.41 (0.92–2.15)	0.114
ECOG 2	1.80 (1.01–3.20)	0.045	2.95 (1.55–5.63)	0.001
ECOG 3	3.39 (1.23–9.36)	0.018	2.73 (0.84–8.87)	0.096
Tumor size > 2.5 cm	1.07 (0.75–1.52)	0.705		
CA19.9 > 37	1.02 (0.62–1.69)	0.932		
Lymph node status (N) *				
N0	Reference		Reference	
N1	1.72 (1.27–2.33)	0.001	1.92 (1.29–2.85)	0.001
N2	1.97 (1.15–3.39)	0.014	1.21 (0.55–2.63)	0.640
Resection margin (R)				
R0	Reference		Reference	
R1	1.34 (1.01–1.78)	0.042	1.14 (0.78–1.67)	0.501
Tumor differentiation				
Well differentiated (G1)	0.67 (0.41–1.07)	0.094	0.69 (0.41–1.17)	0.168
Moderately differentiated (G2)	Reference		Reference	
Poorly differentiated (G3)	2.39 (1.41–4.04)	0.013	1.80 (1.14–2.84)	0.011

* According to AJCC 8th edition, ^$^ *p* < 0.100, ^#^ *p* < 0.05, *p*-values based on complete case analysis.

**Table 3:** Uni- and multivariable Cox regression analysis for recurrence-free survival.

	Univariable Analysis		Multivariable Analysis	
	HR (95% CI)	*p*-Value ^$^	HR (95% CI)	*p*-Value ^#^
Tumor size > 2.5 cm	1.25 (0.82–1.90)	0.303		
T status (T)				
T1	1.12 (0.56–2.23)	0.754	1.60 (0.61–4.20)	0.336
T2a	0.53 (0.34–0.84)	0.007	0.60 (0.36–1.01)	0.056
T2b	1.12 (0.73–1.73)	0.594	1.27 (0.76–2.12)	0.367
T3	Reference		Reference	
T4	1.01 (0.54–1.86)	0.988	1.04 (0.52–2.11)	0.908
Lymph node status (N) *				
N0	Reference		Reference	
N1	1.72 (1.20–2.47)	0.003	2.62 (1.68–4.08)	<0.001
N2	1.86 (0.99–3.50)	0.055	1.249 (0.70–3.17)	0.303
Resection margin (R)				
R0	Reference			
R1	0.99 (0.71–1.38)	0.940		
Tumor differentiation				
Well differentiated (G1)	0.98 (0.57–1.70)	0.946	0.98 (0.57–1.70)	0.416
Moderately differentiated (G2)	Reference		Reference	
Poorly differentiated (G3)	1.65 (1.05–2.58)	0.029	1.65 (1.05–2.58)	0.001

* According to AJCC 8th, ^$^ *p* < 0.100, ^#^ *p* < 0.05, *p*-values based on complete case analysis.

## Data Availability

Data were provided by the ENSCCA Registry. The data presented in this study are available on request from J.M.B.

## Supplementary Materials

The following supporting information can be downloaded at: https://www.mdpi.com/article/10.3390/cancers14102389/s1, Supplementary Table S1: Factors independently associated with positive lymph nodes on uni- and multivariable logistic regression analysis.
