# Supplementary material for: Impact of Positive Lymph Nodes and Resection Margin Status on the Overall Survival of Patients with Resected Perihilar Cholangiocarcinoma: The ENSCCA Registry
Source: Cancers (Basel). 2022 May 12;14(10):2389. doi: 10.3390/cancers14102389 (PMC9140174; doi:10.3390/cancers14102389)
Supplement: Supplementary file 1 [file cancers-14-02389-s001.zip › cancers-1675253-supplementary.pdf]

Table S1: Factors independently associated with positive lymph nodes on uni and multivariable logistic regression analysis.

	**Univariable analysis**		**Multivariable analysis**	
	OR (95% CI)	*P* value[$]	OR (95% CI)	*P* value[#]
Age at surgery	0.98 (0.96 – 1.00)	**0.102**	0.98 (0.96 – 1.01)	0.108
Tumor size >2.5cm	1.62 (0.92 – 2.85)	**0.097**	1.56 (0.88 – 2.76)	0.129
ECOG performance status				
ECOG 0	Reference			
ECOG 1	1.20 (0.68 – 2.13)	0.528		
ECOG 2	0.85 (0.28 – 2.56)	0.774		
ECOG 3	0.68 (0.07 – 6.72)	0.742		
Tumor differentiation				
Well differentiated (G1)	0.62 (0.29 – 1.33)	0.221		
Moderately differentiated (G2)	Reference			
Poorly differentiated (G3)	0.72 (0.38 – 1.36)	0.311		
